# Neural Mechanisms of Autonomic Dysfunction in Neurological Diseases

**DOI:** 10.1155/2017/2050191

**Published:** 2017-12-27

**Authors:** De-Pei Li, Yu-Long Li, Jianhua Li, Sheng Wang

**Affiliations:** ^1^Department of Critical Care, The University of Texas, MD Anderson Cancer Center, Houston, TX, USA; ^2^Department of Emergency Medicine, University of Nebraska Medical Center, Omaha, NE, USA; ^3^Department of Medicine, Penn State College of Medicine, Hershey, PA, USA; ^4^Department of Physiology, Hebei Medical University, Shijiazhuang, Hebei, China

Autonomic nervous system innervates internal organs and plays a crucial role in maintaining hemostasis of cardiovascular, respiratory, energy metabolism, balance of water, electrolyte, and body fluid, and so forth. Dysfunction of the autonomic nervous system is associated with many neurological diseases including neurogenic hypertension, stroke, Alzheimer's disease, Parkinson's disease, and depression. Autonomic dysfunction is also observed in metabolic diseases such as diabetes, obesity, and metabolic syndrome. Many efforts have been made to elucidate the neuronal mechanisms underlying dysautonomia of the cardiovascular system such as hypertension [1]. Overactivation of the sympathetic nervous system is associated with several types of hypertension such as essential hypertension, salt-sensitive hypertension, and obesity-related hypertension, as well as renal hypertension [1, 2]. It has been shown that presympathetic neurons in autonomic nuclei in the hypothalamus and rostral ventrolateral medulla provide excitatory drive to sympathetic outflow in hypertension. Thus, the neural plasticity in these presympathetic neurons and related factors that regulate the excitability of these neurons are crucially important to affect blood pressure and sympathetic vasomotor tone. In this special issue of “Neural Mechanisms of Autonomic Dysfunction in Neurological Diseases,” studies addressed brain stem and hypothalamus mechanisms involved in the regulation of sympathetic outflow and blood pressure.

H. Zheng et al. studied hypothalamic mechanisms in the context of sympathetic activation in hypertension related to obesity or type II diabetes mellitus. They reported that the expression levels of leptin receptor and *N*-methyl-*D*-aspartate receptor (NMDAR) subunit NR1 in the hypothalamus were significantly elevated in a rat model of type II diabetes mellitus induced by high-fat diet and low-dose streptozotocin. They also found that leptin receptors interact with NMDARs in the regulation of sympathetic outflow. The mechanisms associated with the development of hypertension identified in this study may contribute to the elevated sympathetic outflow and provide a potential target to develop a novel treatment for hypertension in type II diabetes mellitus.

A variety of factors may lead to autonomic dysfunction involved in the overactivation of the sympathetic nervous system including diet ingredients and environmental factors. M.-F. Zhong et al. reported in this special issue that homocysteine, a dietary amino acid, in the rostral ventrolateral medulla in the brain stem increased blood pressure and sympathetic outflow. This observed response is mediated by an induction of oxidative stress in the autonomic brain nucleus. A. D. Chapp et al. reported that high-salt diet was an independent risk factor for the development of salt-sensitive hypertension through suppressing small conductance calcium-activated potassium channels and subsequently increasing excitability of presympathetic neurons in the hypothalamus. These findings could significantly improve our understanding of the role of dilatory ingredients in the development of dysautonomia in hypertension. Glutamate is a major excitatory neurotransmitter in the vertebrate nervous system, and glutamatergic synaptic inputs innervate the presympathetic neurons located in the autonomic nucleus in the brain stem and hypothalamus. Enhancement of these excitatory glutamatergic inputs can result in sympathetic overactivation in various animal models of hypertension [1]. W. Wang et al. reported that glutamatergic inputs to the rostral ventrolateral medulla in the brain stem were enhanced, contributing to neuropathic pain-induced high blood pressure.

The purpose of this special issue is to publish findings focusing on neural plasticity of dysautonomia in neurological diseases. Although most of the accepted manuscripts reported neural plasticity in dysfunction of the autonomic nervous system in the regulation of sympathetic activity and blood pressure, these mechanisms may also apply to autonomic dysfunction during other neurological diseases. Therefore, the studies compiled in this special issue may promote research activities of neural mechanisms underlying autonomic dysfunction in neurological diseases.
